# Transcriptomic Analysis Reveals the Metabolic Mechanism of L-Ascorbic Acid in *Ziziphus jujuba* Mill.

**DOI:** 10.3389/fpls.2016.00122

**Published:** 2016-02-15

**Authors:** Chunmei Zhang, Jian Huang, Xingang Li

**Affiliations:** Comprehensive Laboratory of Forest for Shaanxi Province, College of Forestry, Northwest A&F UniversityYangling, China

**Keywords:** *Ziziphus jujuba* Mill., L-ascorbic acid, gene expression, ascorbate biosynthesis, ascorbate recycling

## Abstract

Chinese jujube (*Ziziphus jujuba* Mill.) is the most economically important member of the Rhamnaceae family and contains a high concentration of ascorbic acid (AsA). To explore the metabolic mechanism of AsA accumulation, we investigated the abundance of AsA in the fruit development stages, the leaf and flower of *Z. jujuba* cv Junzao, and the mature fruit of one type of wild jujube (*Z. jujuba* var. *spinosa* Hu, Yanchuan sour jujube). And the expression patterns of genes involved in AsA biosynthesis, degradation, and recycling were analyzed. The result showed that AsA biosynthesis during early fruit development (the enlargement stage) is the main reason for jujube high accumulation. The L-galactose pathway plays a predominant role in the biosynthesis of AsA during jujube fruit development, and the genes *GMP1, GME1, GGP*, and *GaLDH* involved in the determination of AsA concentration during fruit development and in different genotypes; the myo-inositol pathway along with the genes *GME*2 and *GMP*2 in the L-galactose pathway play a compensatory role in maintaining AsA accumulation during the ripening stage. These findings enhance our understanding of the molecular mechanism in regulating AsA accumulation for jujube.

## Introduction

Ascorbic acid (AsA), also called vitamin C, is an important antioxidant that plays important roles in several plant biological processes including defense mechanisms, cell division, and photosynthesis (Smirnoff, 2011). AsA, which cannot be synthesized by human body, is an important dietary supplement for humans. Fruits and vegetables synthesize relative high levels of AsA, however, AsA content varies markedly among plant tissues and plant species.

The AsA content of a tissue is determined by its biosynthesis and recycling. Four biosynthetic pathways have been reported in plants—the L-galactose pathway (Wheeler et al., 1998), the myo-inositol pathway (Lorence et al., 2004), the galacturonate pathway (Agius et al., 2003), and the L-gulose pathway (Wolucka and Van Montagu, 2003). In AsA recycling, AsA is produced by a reduction of oxidized forms of AsA, including monodehydroascorbate and dehydroascorbate which also contributes to determination of the AsA content (Alós et al., 2013).

**Figure 1:**
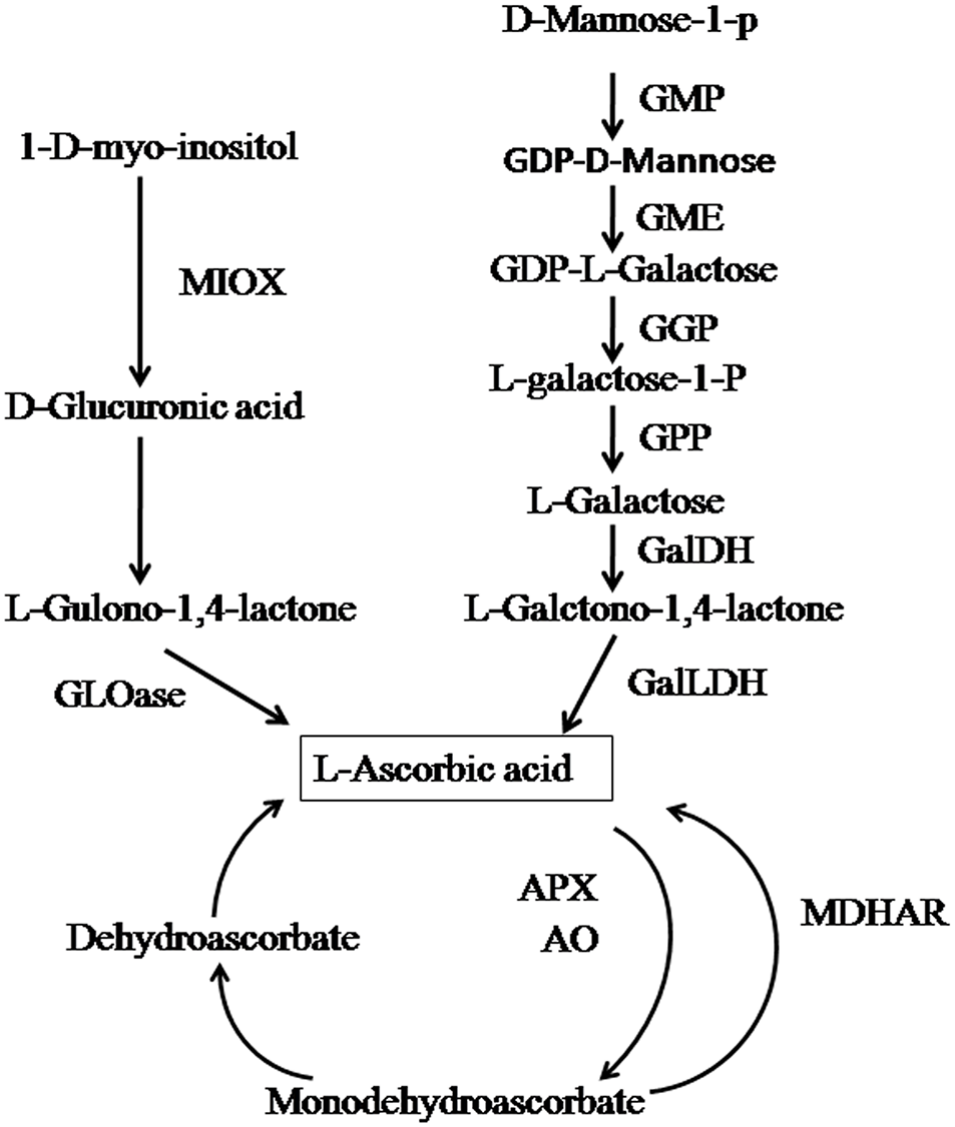
**AsA biosynthesis, degradation, and recycling pathways**. The enzymes catalyzing the reactions are: GDP-d-mannose pyrophosphorylase (*GMP*), GDP-mannose-3′-5′-epimerase(*GME*), GDP-l-galactose transferase (*GGP*), l-galactose-1-phosphate phosphatase (*GPP*), l-galactose dehydrogenase (*GalDH*), l-galactono-1,4-lactone dehydrogenase (*GalLDH*), myo-inositol oxygenase (*MIOX*), ascorbate peroxidase (*APX*), ascorbate oxidase (*AO*), dehydroascorbate reductase (*DHAR*), monodehydroascorbate reductase (*MDHAR*). Adapted from Liu et al. (2014).

Chinese jujube (*Ziziphus jujuba* Mill.) is the most economically important member of the family Rhamnaceae due to its important nutritional and medicinal properties (Qu and Wang, 1993). As a fruit native to China (Qu and Wang, 1993), it has been cultivated for 3000 years (Liu et al., 2013), with a cultivation area of more than 2 million Ha (Liu et al., 2013), and is widely distributed in more than 47 countries. As an important economic fruit tree, it contains a variety of phytochemicals beneficial for human health. Particularly, the concentration of AsA in jujube fruit is among the highest of all fruits including kiwifruit, and can reach 1000 mg/100 g fresh weight (FW) (Liu and Wang, 2009). In recent years, intensive efforts have focused on the metabolism of AsA in many important fruits; e.g., kiwifruit, citrus fruits, tomato, and roxburgh rose (Li et al., 2010; Mellidou et al., 2012a,b; Huang et al., 2014). In jujube, however, the characteristics of AsA accumulation in fruit and among tissues and species are unknown. A genome-sequencing analysis of *Z. jujuba* cv. Dongzao (Liu et al., 2014) demonstrated the presence of two biosynthetic pathways (the L-galactose and myo-inositol pathways) in jujube (Figure 1). However, the key genes or gene families in AsA synthesis, degradation, and recycling, and their relative contributions to AsA concentrations at the various fruit developmental stages and in various plant tissues, have not been well understood.

To increase our understanding of AsA production in jujube fruit, the changes in the concentrations of AsA and related metabolites were systematically analyzed. The gene expression patterns during fruit development and among the leaf, flower, and the mature fruit of one wild species of jujube (*Z. jujuba* var. *spinosa* Hu) were evaluated. These findings will be beneficial for plant breeding strategies in improving AsA content.

## Materials and methods

### Plant materials and sample preparation

The Chinese jujube cultivar “Junzao” (*Z. jujuba* cv. Junzao) was grown in the Experimental Station of Jujube of Northwest A&F University in Qingjian, Shaanxi, China. Fruits at all developmental stages [young fruits at 10 days after the anthesis (DAA), two enlargement fruits at 30 and 50 DAA, white mature fruits at 80 DAA, half-red fruits at 100 DAA, and full-red fruits at 110 DAA] were collected, among which, young and enlargement fruits belong to the early developmental stages. The fruit begins to ripen at the white mature stage. We also harvested leaves and flowers in florescence. Mature fruit of one type of wild jujube, “Yanchuan sour jujube” (*Z. jujuba* var. *spinosa* Hu cv Yanchuansuanzao), was also harvested. The fruit was cut into small pieces, immediately frozen in liquid nitrogen and stored at −80°C. Other sampled tissues were directly frozen in liquid nitrogen and stored at −80°C.

### HPLC analysis of AsA

AsA was extracted and determined essentially as described in Huang et al. (2014). Frozen tissue (0.5 g) was added to 3 ml of 0.2% metaphosphoric acid and directly ground using a precooled mortar. The homogenate was centrifuged for 15 min at 12,000 rpm at 4°C. The supernatant was diluted with 0.2% metaphosphoric acid to 10 ml and used for AsA determination. To determine the total AsA level, the method described by Gökmen et al. (2000) was used. Thus, a 1000 μl aliquot of supernatant was incubated for 4 h in the dark with 10 μl of 200 mM dithiothreitol (DTT). Dehydroascorbic acid (DHA) content was evaluated as the difference between total and reduced AsA levels.

AsA was evaluated using a high performance liquid chromatography (HPLC) system with a photodiode array detector, Chromeleon software (Dinex), and a reverse C18 column. The mobile phase was composed of 15% methanol and 85% metaphosphoric acid aqueous solution, pH 2.5. The flux was set to 1 ml/min and the injection volume was 10 μl. The column temperature was set at 35°C. Spectra were acquired at wavelengths between 200 and 400 nm and AsA quantification was performed at 243 nm. An ascorbic acid standard curve was generated using standards with concentrations of 5–500 μg/mL using the same method. All samples were extracted and analyzed in triplicate.

### RNA extraction, cDNA synthesis, and selection of genes involved in AsA metabolism

Total RNA was extracted using a TaKaRa MiniBEST Plant RNA Extraction Kit and treated with the Recombinant DNase I included in the kit following the manufacturer's instructions. The integrity of the total RNA was estimated using 1.5% agarose gel electrophoresis. RNA was quantified using a NanoDrop20000 (Thermo Scientific). Total RNA was reverse-transcribed using a PrimeScript RT Reagent Kit (TaKaRa).

The genes involved in AsA metabolism were identified from the whole-genome sequence of *Z. jujuba* Mill. cv. Junzao (accession number: SAMN04349627, Supplementary Tables 1, 2). These sequences were aligned by ClustalW with MEGA5. The phylogenetic tree was constructed using the Neighbor-Joining method and the phylogeny test was based on the bootstrap method with 1000 replications. RNA-seq reads for transcriptome analysis were got from NCBI database with the accession numbers SRS1237017 (leaves of Junzao), SRS1237016 (mature fruit of Junzao), SRS1237011 (flower of Junzao), and SRS1237074 (mature fruit of Yanchuan sour jujube). The data quality of the samples was shown in Supplementary Table 3. The normalized read counts [RPKM (Mortazavi et al., 2008)—reads per kb per million] were used as the expression level for each gene.

### Gene expression analysis by real-time PCR

Quantitative real-time RT-PCR (qRT-PCR) was performed using a SYBR Premix Ex Taq Kit (TaKaRa) on a Bio-Rad IQ5 instrument. The PCR mix contained 2 μl of cDNA, 10 μl of SYBR Green Master Mix, 0.8 μl of 10 μM primer F, and 0.8 μl of 10 μM primer R in a final volume of 20 μl. Primers were designed using Primer3 software (Rozen and Skaletsky, 2000). Primer sequences of the qRT-PCR products were obtained from the genome sequence of *Z. jujuba* var. Junzao (Supplementary Table 1). The PCR protocol was as follows: 95°C for 3 min, followed by 40 cycles at 95°C for 5 s and 56°C for 30 s. To confirm primer specificity, the melting curve was analyzed at 60–95°C for 5 s after 40 cycles. *UBQ* was selected as the reference gene according to Zhang et al. (2015). A standard curve was generated using three replicates of each concentration to calculate the amplification efficiency [*E* = 10^(−1∕slope)^] and correlation coefficient (*R*^2^) of each primer (Bustin et al., 2009). Gene expression levels were calculated using the formula 2^∧^- delta CT according to Livak and Schmittgen (2001). The result of each gene for each sample was the average of three biological and technical repetitions, and the mean with standard error was calculated by software SPSS17.0. Duncan's multiple test (5%) was used to evaluate the differences among tissues.

## Results

### Changes in AsA concentration during jujube fruit development

**Figure 2:**
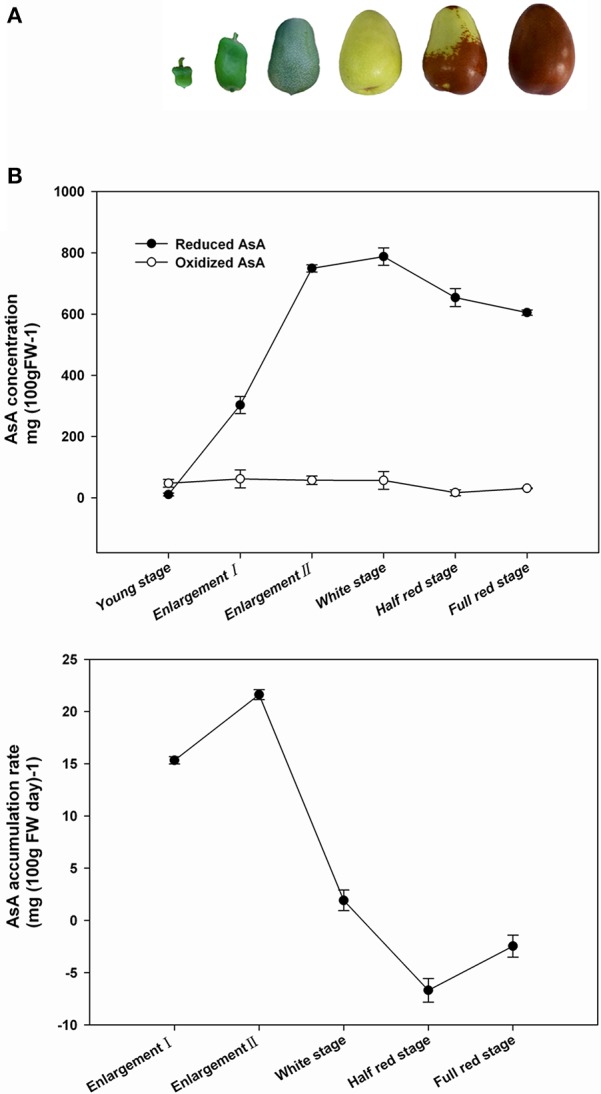
**Phenotype of ***Ziziphus jujuba*** cv. Junzao fruits during development and ripening**. **(A)** AsA content and accumulation rate **(B)** in jujube fruits according to developmental stages.

AsA accumulation in young fruit, enlargement fruit (two stages), white mature fruit, half red fruit, and full red fruit of Junzao jujube was evaluated (Figure 2B). Reduced AsA concentration was low at the young fruit stage (10.5 mg/100 g FW), and was lower than that of oxidized AsA (47.3 mg/100 g FW), but rapidly increased to 749.2 mg/100 g FW during the fruit enlargement stage, and peaked (787.8 mg/100 g FW) at the white mature stage. The concentration of AsA decreased slightly upon fruit ripening, which corresponds to the transition from green to red fruit (Figure 2A). Oxidized AsA concentration remained relatively stable during fruit development but decreased slowly in half-red stage, and increased again in fully matured (full-red stage) fruit. The rate of AsA accumulation per day was calculated for each developmental stage (Figure 2B). The highest AsA accumulation rate in fruit was 22 mg day^−1^ per 100 g FW during the fruit enlargement stage. The accumulation rate was less than zero following the white mature stage.

### AsA redox state in the fruit, leaf, and flower of junzao and fruit of Yanchuan sour jujube

**Figure 3:**
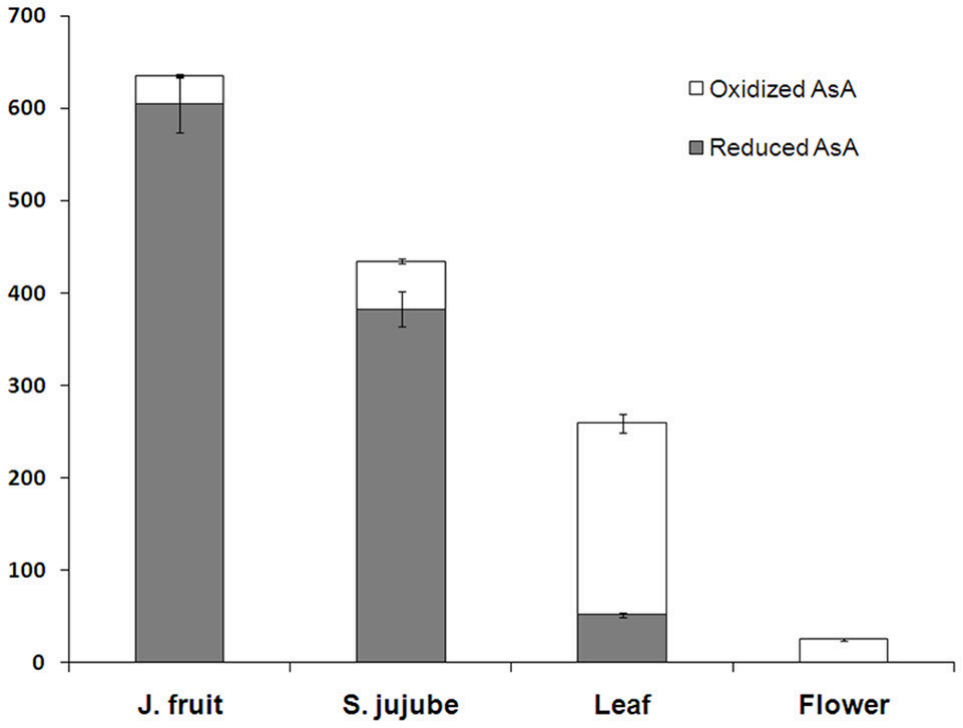
**AsA concentration of mature fruit, leaf, and flower**. Data are expressed as the means ± standard deviation (SD) of three replicates. J. fruit (jujube fruit), S. fruit (Sour jujube fruit).

Reduced and oxidized AsA concentrations were also measured in leaf, flower of Junzao and the mature fruit of Yanchuan sour jujube (Figure 3). Comparison of AsA concentrations in Junzao mature fruit, leaf, and flower showed that the fruit contained the highest reduced AsA levels (604.7 mg/100 g) and low levels of oxidized AsA (30.9 mg/100 g). The leaf contained high levels of oxidized AsA (207.1 mg/100 g) and low levels of reduced AsA (52.2 mg/100 g). The flower contained the lowest total AsA level (25 mg/100 g) and reduced AsA was not detected. Compared with Junzao, the fruit of Yanchuan sour jujube had a lower reduced AsA concentration (382.8 mg/100 g) and higher oxidized AsA concentration (52.2 mg/100 g).

### Selection of genes involved in AsA metabolism for analysis by qRT-PCR

To evaluate the AsA biosynthesis and recycling metabolic pathways in jujube, the expression patterns of several important genes associated with AsA metabolism were analyzed. A total of 46 genes involved in AsA metabolism were identified in the whole-genome sequence of *Z. jujuba* Mill. cv. Junzao (accession number: SAMN04349627). The biosynthetic genes included four isoforms of *GDP-mannose pyrophosphorylase* (*GMP*), two isoforms of *GDP-mannose-3*′*-5*′*-epimerase* (*GME*), two isoforms of *GDP-l-galactose phosphorylase* (*GGP*), two isoforms of *l-galactose-1-phosphate phosphatase* (*GPP*), two isoforms of *l-galactose dehydrogenase* (*GalDH*), and *l-galac-tono-1,4-lactone dehydrogenase* (*GalLDH*), which are involved in the L-galactose pathway, and four isoforms of *myo-inositol oxygenase* (*MIOX*), which are involved in the myo-inositol pathway. AsA degradation genes included 17 isoforms of *ascorbate oxidase* (*AO*) and eight isoforms of *ascorbate peroxidase* (*APX*). In the recycling pathway, four genes were detected: two isoforms of *monodehydroascorbate reductase* (*MDHAR*) and two of *dehydroascorbate reductase* (*DHAR*). As shown in Supplementary Figure, in accordance with the current literature, nine subfamilies were identified except the *DHAR*s. The same enzyme clustered together except the DHARs. In the phylogenetic tree of the *AO* family, the results showed that the 17 isoforms of *AO*s were categorized into three subgroups, *AO*1–*AO*7 were clustered into a clade, *AO*13 and *AO*17 were clustered into another clade, and the others formed an additional clade. In the phylogenetic tree of *APX* family, *APX*6 had the farthest genetic relationship to the other ones, and *APX*5 and *APX*7 had the same sequence.

Since some of these genes were not expressed or expressed at low levels according to the RNA sequencing data-the normalized read counts (< 10; Supplementary Table 2), they were excluded from further analyses. In total, 10 genes involved in biosynthesis (two isoforms of *GMP, GME*, and *MIOX*, and one of *GGP, GaLDH, GalLDH*, and *GPP*) and 12 genes involved in dehydroascorbate and recycling (five isoforms of *AO*, four of *APX*, two of *MDHAR*, and one of *DHAR*) were analyzed by qRT-PCR. The *E*-values of the 22 reference genes varied from 97 to 110%, and the *R*^2^-values from 0.994 to 1.000 (Supplementary Table 1).

### Expression of genes involved in AsA biosynthesis according to fruit developmental stage

**Figure 4:**
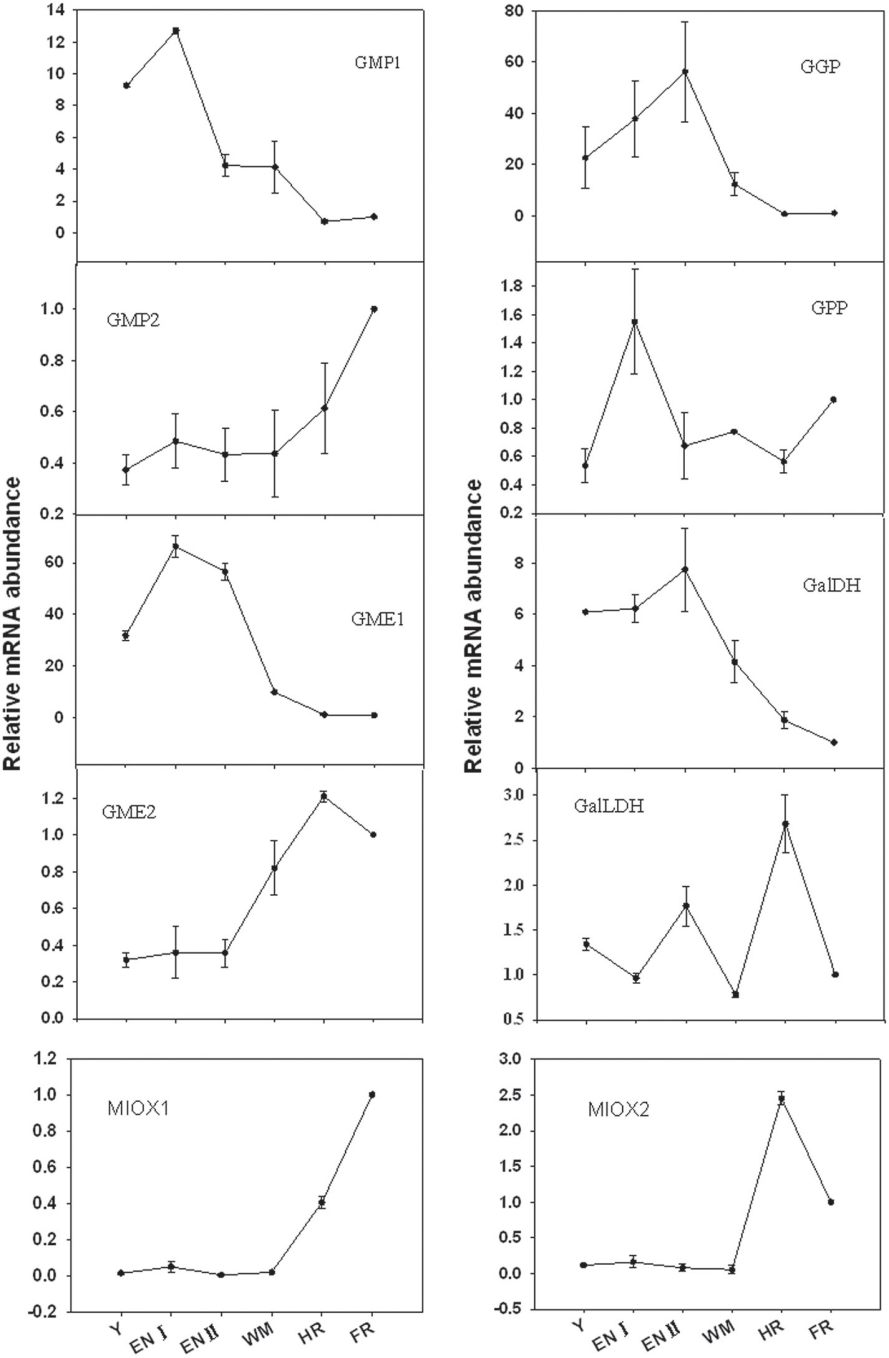
**Relative expression levels of genes involved in the L-galactose and myo-inositol AsA biosynthetic pathways in fruit of ***Z. jujuba*** Mill**. **(***Y***, young stage; ***EN*** I, enlargement I stage; ***EN*** II, enlargement II stage; ***WM***, white mature stage; ***HR***, half-red stage; ***FR***, full-red stage)**. Results are normalized to the expression value of full-red fruit, which was set to 1. Data are expressed as the means ± SD of three replicates.

The expression of 10 genes involved in biosynthesis was evaluated in Junzao fruit at various developmental stages (Figure 4). Expression levels were normalized to the value in full-red fruit. The genes exhibited varying expression patterns. In the L-galactose pathway, *GMP1, GME1, GGP*, and *GaLDH* were expressed at relatively high levels at the young fruit stage, and at the highest levels during the enlargement stage (I or II). Their expression levels then progressively declined until fruit ripening. In contrast, expression levels of *GMP2* and *GME2* were low at the early developmental stages and underwent an increment (two to four folds) at the mature stages. Other genes in this pathway include *GPP*, the expression level of which was higher at the enlargement stage and lower in the other stages, and *GalLDH*, the expression level of which was highest at the half-red stage followed by the enlargement stage and was relatively low in the other stages. In the myo-inositol pathway, two isoforms of *MIOX* were minimally expressed during the early stages of fruit development and increased at the half-red stage. The expression of *MIOX1* was highest at the full-red stage; in contrast, *MIOX2* expression decreased during the full-red stage.

### Expression of genes involved in AsA degradation and recycling according to fruit developmental stage

**Figure 5:**
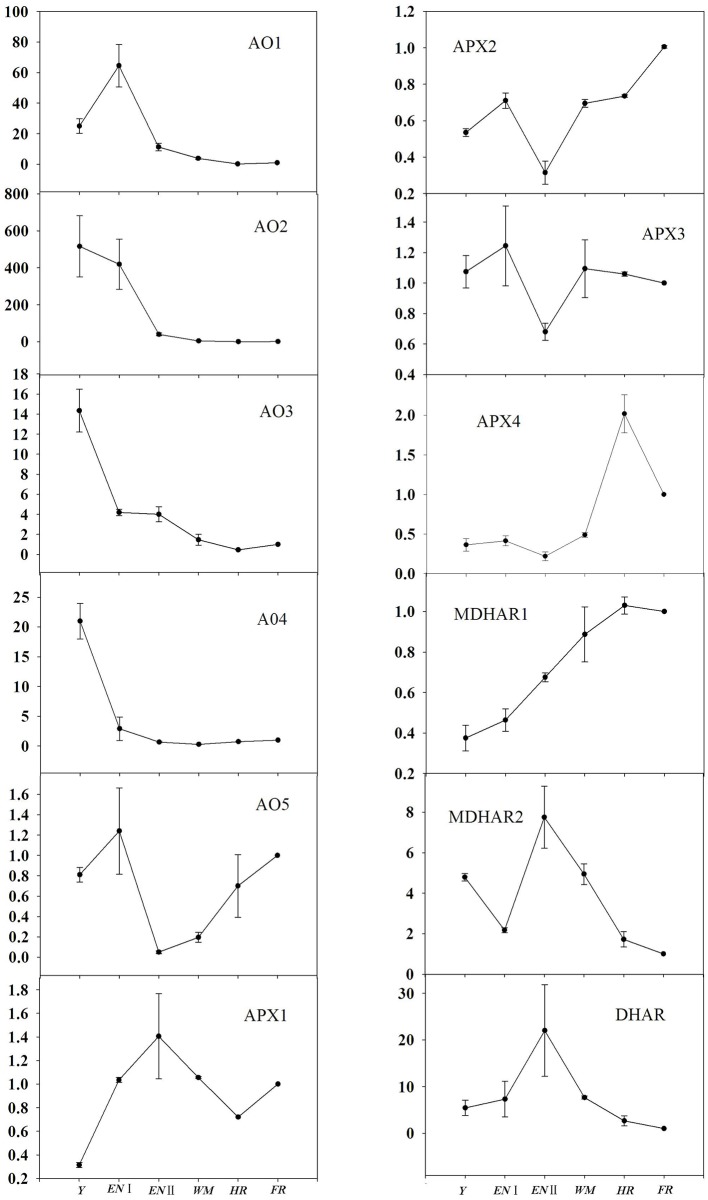
**Relative expression levels of genes involved in AsA degradation and recycling in fruit of ***Z. jujuba*** Mill**. **(***Y***, young stage; ***EN*** I, enlargement I stage; ***EN*** II, enlargement II stage; ***WM***, white mature stage; ***HR***, half-red stage; ***FR***, full-red stage)**. Results are normalized to the expression value of full-red fruit, which was set to 1. Data are expressed as the means ± SD of three replicates.

With regard to AsA degradation, the expression profiles of *AO* and *APX* were analyzed (Figure 5). *AO* genes were highly expressed during early fruit development (*AO2, AO3*, and *AO4* at the young fruit stage, and *AO1* and *AO5* at the enlargement stage). *AO2, AO3*, and *AO4* expression decreased markedly in the enlargement I stage, as did that of *AO1* in the enlargement II stage and remained stable thereafter/from the white mature stage. The *AO5* expression profile was similar to that of *APX2*; their expression was lowest at the enlargement II stage and increased gradually as fruit ripening progressed. *APX1* was expressed at a low level in young fruit and fluctuated as fruit development progressed, while *APX3* expression was lowest at the enlargement II stage. In contrast, *APX4* expression was stable during the first four developmental stages, then increased from the white to the half-red stage and decreased slightly at the full-red stage.

Regarding AsA recycling, *MDHAR1* expression increased as fruit development progressed, while *MHAR2* and *DHAR* expression peaked at the enlargement II stage and decreased gradually thereafter.

### Expression of genes involved in AsA biosynthesis and recycling in leaf, flower, mature fruit of junzao, and mature fruit of Yanchuan sour jujube

**Figure 6:**
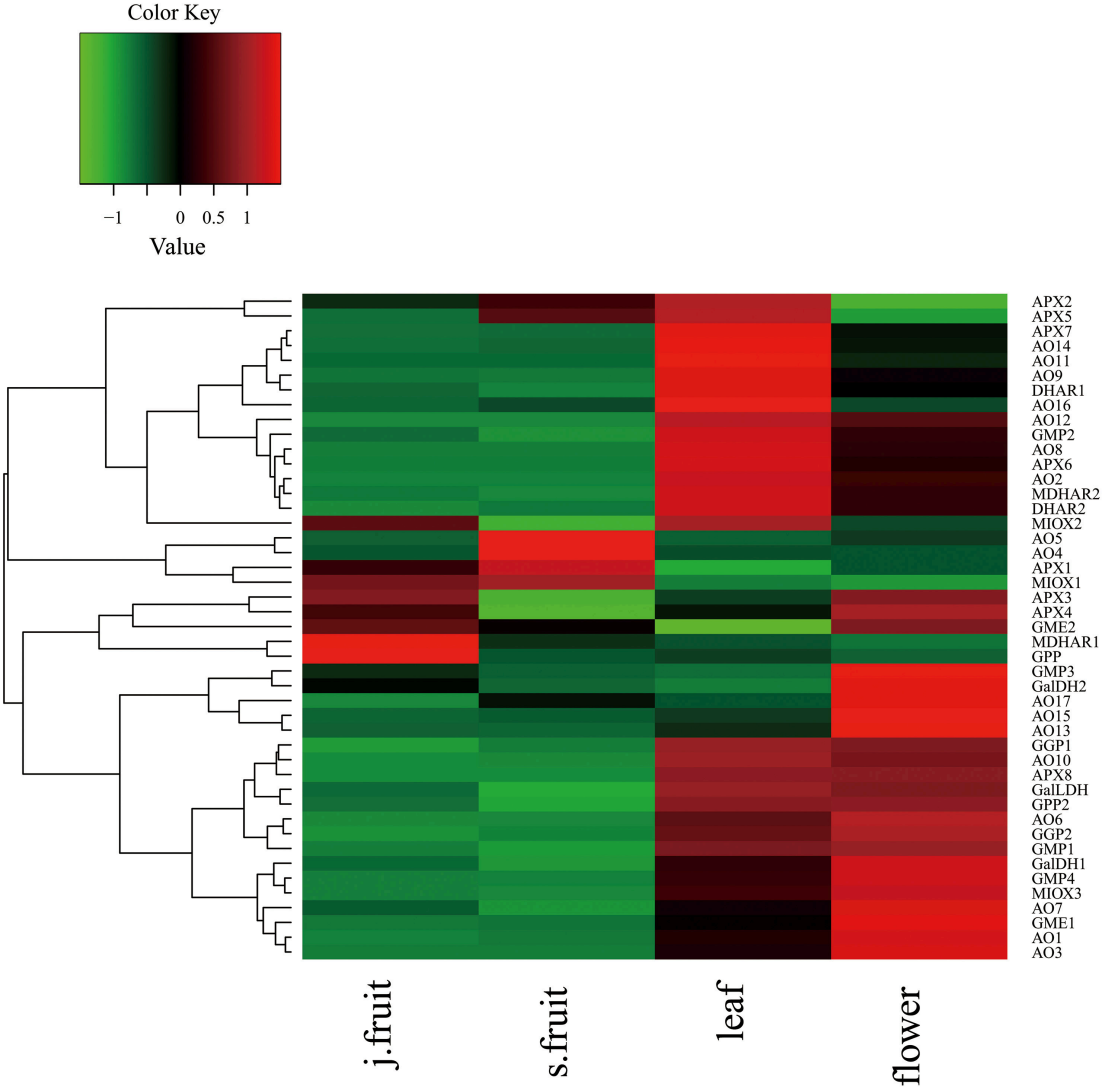
**Heatmap of RNA-Seq data for genes involved in AsA biosynthetic, degradation, and recycling in Junzao jujube fruit (j.fruit), leaf, flower, and Yanchuan sour jujube fruit (s.fruit)**.

To explore AsA metabolism in the various organs of jujube, we analyzed the expression of genes related to AsA metabolism from the RNA-Seq data (Supplementary Table 2 and Figure 6). The expression of most genes—including those related to AsA biosynthesis and degradation—in the leaf and flower was higher than in the fruit. When compared with Junzao, the genes up-regulated in Yanchuan sour jujube fruit were those related to AsA degradation, such as *AO10, AO17, APX2, AO4, APX6, AO5*, and *AO15*; those down-regulated included the biosynthesis-related genes *GPP, GMP1, GME2*, and *GalLDH*, and the recycling-related gene *MDHAR1*.

**Figure 7:**
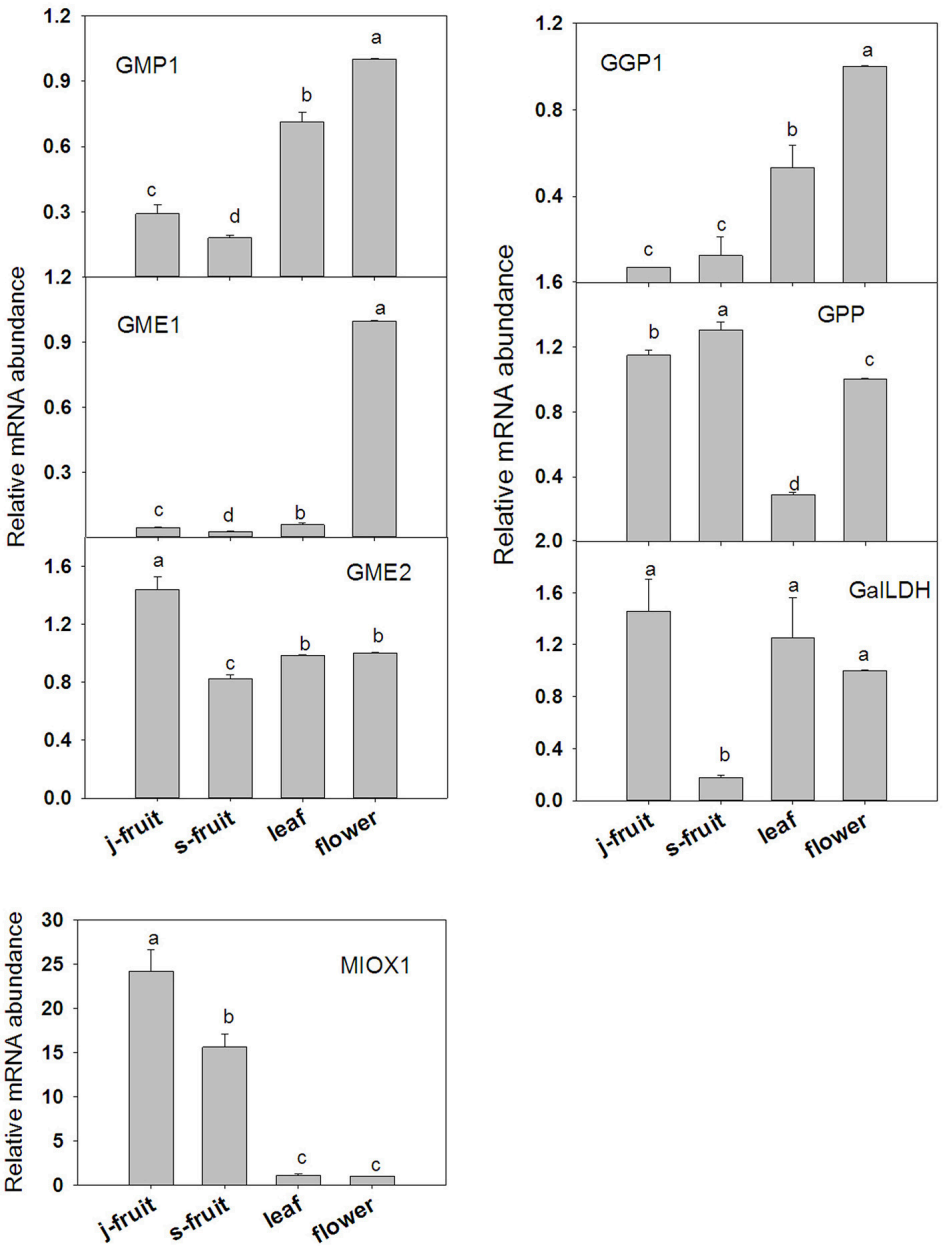
**Relative expression levels of selected genes involved in the AsA biosynthetic in Junzao jujube fruit (j.fruit), leaf, flower, and Yanchuan sour jujube fruit (s.fruit)**. Results are normalized to the expression value of leaf, which was set to 1. Data are expressed as the means ± SD of three replicates. Different lowercase letters indicate significant differences with each other based on Duncan's multiple test (5%).

**Figure 8:**
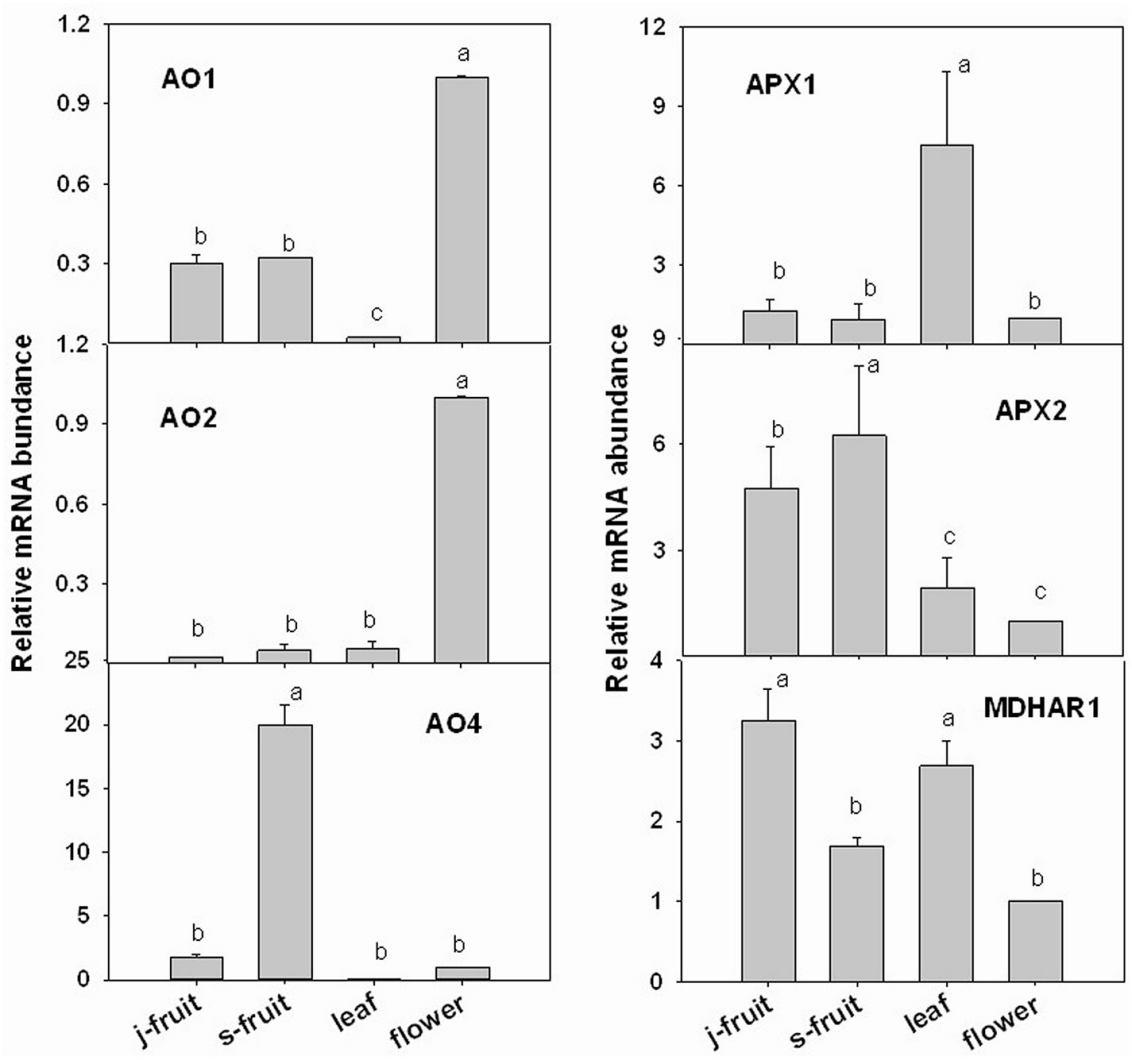
**Relative expression levels of selected genes involved in the AsA degradation and recycling pathways in Junzao jujube fruit (j.fruit), leaf, flower, and Yanchuan sour jujube fruit (s.fruit)**. Results are normalized to the expression value of leaf, which was set to 1. Data are expressed as the means ± SD of three replicates. Different lowercase letters indicate significant differences with each other based on Duncan's multiple test (5%).

To validate the transcriptome RNA-Seq results, we conducted a qRT-PCR analysis of several key genes—*GMP1, GME1, GME2, GGP1, GPP, GalLDH, MIOX1, MDHAR1, APX1, APX2, AO1, AO2*, and *AO4* (Figures 7, 8). Expression levels were normalized to those in flowers. In the AsA biosynthetic pathway, expression of genes *GMP1, GME1, GME2, GalLDH*, and *MIOX1* was significantly higher in Junzao than Yanchuan sour jujube (Figure 7). Expression of *MDHAR1*, which is important in AsA recycling, was also up-regulated in Junzao (Figure 7). In contrast, expression of the degradation-related genes *AO4* and *APX2* was lower in Junzao than in Yanchuan sour jujube (Figure 8). When compared with fruit, expression of genes *GMP1, GGP1*, and *GME1* in leaf and flower, *AO1* and *AO2* in flower, and *APX1* in leaf was up-regulated, which is consistent with the RNA-Seq data. Thus, the expression levels obtained using the two methods were largely in agreement, with the exception of *MIOX1* and *GPP*.

## Discussion

### AsA accumulation according to fruit developmental stage

Chinese jujube is recognized as one of the most important fruits with high vitamin C content (Liu and Wang, 2009). Our results showed that AsA accumulated mainly during the enlargement stage and then decreased slightly from the white mature stage until the full-red stage. Similar AsA change has also been reported in kiwifruit during the early stages of fruit development (Li et al., 2010). By contrast, tomato synthesizes AsA primarily during fruit ripening after the turning stage (Ioannidi et al., 2009). Of peach, AsA concentration declines progressively during development (Imai et al., 2009). Some other fruit species accumulate AsA continually during their development (Alós et al., 2013, 2014). As one of the most important factors involved in rapid cell proliferation (Kato and Esaka, 2000), a rapid increase in AsA level would enhance jujube fruit enlargement during the early developmental stages. The decrease in AsA accumulation in the final stage of fruit ripening may be explained by the transformation of chloroplasts to chromoplasts in the peel, which corresponds to the loss of chlorophyll.

### AsA metabolism pathways in jujube and other fruit species

Two biosynthetic pathways were annotated in Junzao (dried variety): the L-galactose and myo-inositol pathways. This result is consistent with the genome sequence of Dongzao (fresh variety; Liu et al., 2014), indicating the consistent biosynthetic pathways in the dried and fresh varieties of jujube. However, AsA metabolism pathways vary among plant species. Some fruits, such as grape, tomato, and pepper, have three AsA biosynthetic pathways (Melino et al., 2009; Badejo et al., 2012; Alós et al., 2013): the L-galactose, myo-inositol, and D-galacturonic acid pathways. However, the fruit of jujube contained a higher AsA concentration than did these fruits. Therefore, the AsA levels in different fruit species might not be related to the number of pathways, but may instead be associated with the metabolic or recycling capacity of the pathways in each species (Liu et al., 2014).

### Expression of genes involved in AsA biosynthesis during fruit development

Our results indicate that the increase in AsA accumulation rate is associated with the induction of *GMP1, GME1, GPP*, and *GaLDH* expression before the full-red stage. Expression of *GGP* thought to be the key regulatory gene of AsA accumulation via the L-galactose pathway in many fruits (Bulley et al., 2012; Mellidou et al., 2012a,b) was also up-regulated rapidly when the AsA accumulation rate increased rapidly. These findings indicate that the L-galactose AsA biosynthetic pathway may play a predominant role during jujube fruit development, and that *GMP1, GME1, GGP, GPP*, and *GaLDH* genes are involved in the determination of AsA concentration during fruit development.

The myo-inositol pathway may play a key role in maintaining AsA accumulation at the ripening stage, since the two *MIOX* isoforms, which expressed at negligible levels prior, were up-regulated from the half-red stage. Besides, *GME*2 and *GMP*2 which belong to the L-galactose pathway were also up-regulated during the mature stages. Of tomato, *MIOX* was highly expressed at the early stage of fruit development but was undetected in the maturation stage (Ioannidi et al., 2009).

The main biosynthetic pathways might differ among fruits and developmental stages. For example, in immature grape and tomato, the main AsA synthesis pathway is the L-galactose pathway, while in ripe fruits, the D-galacturonic acid pathway predominates (Melino et al., 2009; Badejo et al., 2012). In immature orange fruit, both the L-galactose and myo-inositol pathways contribute to AsA accumulation, at the mature stage the L-galactose pathway predominates (Alós et al., 2014). Our results suggest that, in jujube, the L-galactose pathway is the primary mechanism of AsA synthesis during fruit development, while the myo-inositol pathway along with the L-galactose pathway may play a compensatory role during fruit ripening.

### Expression of genes involved in AsA degradation and recycling during fruit development

AsA concentration is determined not only by its biosynthesis but also by its degradation and recycling. Our results indicate that the expression of most isoforms of *AO* genes is high in young fruit, decreased by several dozen (*AO*1, *AO*3, and *AO*4) or hundred (*AO*2) fold from the young fruit to the enlargement stage, and then remained almost constant thereafter. This *AO* expression pattern has also been reported in other fruits, such as pepper, chestnut rose, tomato, and citrus (Ioannidi et al., 2009; Alós et al., 2013, 2014; Huang et al., 2014). These findings are likely related to the role of *AO* in the regulation of cell division and proliferation in rapidly growing fruit (Kato and Esaka, 2000; Sanmartin et al., 2007). The expression of *APX2* and *APX3* declined to the lowest level during the enlargement II stage, in accordance with the highest AsA accumulation rate. Moreover, the high level of *APX2* and *APX4* expression at the mature stage was consistent with the decline in AsA content. These results suggest a crucial role for *APX* in AsA degradation. In a study of citrus fruit, the highest *APX* expression level also occurred simultaneously with the degradation of AsA during the development and maturation stages (Alós et al., 2014). Therefore, *APX* genes, in particular *APX2, APX3*, and *APX4*, appear to be important for the regulation of AsA concentration via its degradation.

AsA recycling plays an important role in maintaining the high concentration of AsA during fruit development and maturation. In our study, *MDHAR* and *DHAR* were differentially regulated at the various fruit developmental stages. *MDHAR1* would likely contribute to the maintenance of a relatively high AsA content at the fruit ripening stage since its expression increased throughout fruit development. *MDHAR2* and *DHAR* might play important roles in AsA accumulation in the early stages of development, as they were expressed at the highest level in the enlargement II stage. The crucial genes in the recycling pathway vary among species. In strawberry, the variation in fruit AsA content correlates well with the expression of *MDHAR*, but not with that of *DHAR* (Cruz-Rus et al., 2011), while in the chestnut rose (Huang et al., 2014), it is the expression of *DHAR*, instead of *MDHAR*, that is crucial for AsA accumulation. In our study, however, the AsA recycling pathway was regulated by *MDHAR* and *DHAR* in a cooperative manner.

### AsA biosynthesis and recycling in fruit, leaf, and flower

Junzao jujube fruit contained a higher AsA concentration than Yanchuan sour jujube fruit. This could be explained by the high variability among genotypes. Analysis of the expression of AsA biosynthetic and recycling genes revealed that *GMP*1, *GME*1, *GaLDH, GME*2, *MIOX*1, and *MDHAR*1 expression was lower in Yanchuan sour jujube than Junzao jujube. These genes are similar to those that contribute to AsA concentration during fruit development and ripening. These results suggest that the key genes involved in the regulation of AsA concentration during fruit development are identical in fruits of different genotypes.

As the sink organ, the jujube fruit contains a considerably higher AsA concentration than does the leaf and flower. However, jujube leaf and flower exhibit higher biosynthetic and degradation capacities than the fruit. Moreover, the degradation capacity in leaves is in accordance with the higher concentration of oxidized AsA in this organ. The high *AO* and *APX* expression in leaf and flower is also consistent with the antioxidant function of these organs.

## Conclusion

As the most economically important member of the Rhamnaceae family, jujube fruit is abundant in AsA (~1000 mg/100 g; Liu and Wang, 2009), close to the fruits with top AsA concentration, e.g., kiwifruit (*Actinidia eriantha*) and chestnut rose (Rosa roxburghii Tratt; Huang et al., 2014). Therefore, exploring the metabolic mechanism of AsA in jujube is necessary. Our results indicate that, during fruit development, AsA accumulates mainly during the early stage of development (the enlargement stage), and that the fruit contains a considerably higher AsA concentration than the leaf and flower. Two biosynthetic pathways were annotated in Junzao (dried variety): the L-galactose and myo-inositol pathways. The *GMP*1, *GME*1, *GGP, GPP*, and *GaLDH* genes in the L-galactose pathway contribute to AsA concentration in fruit development; and the myo-inositol pathway and the *GMP*2 and *GME*2 in the L-galactose pathway may play a key role in the maintenance of AsA accumulation at the ripening stage. The AsA recycling pathway was regulated by *MDHAR* and *DHAR* in a cooperative manner. The key genes involved in the regulation of AsA concentration during fruit development, and in fruits of different genotypes and different tissues, were similar. The results also indicate that AsA accumulation is regulated by the combined activities of the biosynthetic, degradation, and recycling pathways. In all, the results increase our understanding of AsA metabolic mechanism in the fruit trees with high AsA content and would provide information for plant breeding strategies toward the enhancement of AsA content in jujube and other fruits.

## Author contributions

CZ was responsible for design of the work, analysis, interpretation of data for the work and drafting the work. JH did substantial contributions to the conception and revised it critically for important intellectual content. XL is accountable for all aspects of the work in ensuring that questions related to the accuracy and integrity of any part of the work are appropriately investigated and resolved.

### Conflict of interest statement

The authors declare that the research was conducted in the absence of any commercial or financial relationships that could be construed as a potential conflict of interest.

## Supplementary material

Supplementary Figure **Maximum likelihood phylogeny of the genes involved in AsA biosynthetic, degradation, and recycling**. **(A)** AsA biosynthetic and recycling gene families **(B)** ascorbate oxidase family **(C)** ascorbate peroxidase family.

Supplementary Table 1 **Primer sequences and amplication characteristics of ASA related genes for RT-qPCR analysis**.

Supplementary Table 2 **The expression levels of genes related to AsA metabolism based on the RNA-Seq data**.

Supplementary Table 3 **The data quality of the samples based on RNA sequencing**.
