# Supplementary material for: Transcriptomic Analysis Reveals the Metabolic Mechanism of L-Ascorbic Acid in Ziziphus jujuba Mill
Source: Front Plant Sci. 2016 Feb 15;7:122. doi: 10.3389/fpls.2016.00122 (PMC4753306; doi:10.3389/fpls.2016.00122)
Supplement: Supplementary Figure — Maximum likelihood phylogeny of the genes involved in AsA biosynthetic, degradation, and recycling. (A) AsA biosynthetic and recycling gene families (B) ascorbate oxidase family (C) ascorbate peroxidase family. [file Image1.pdf]

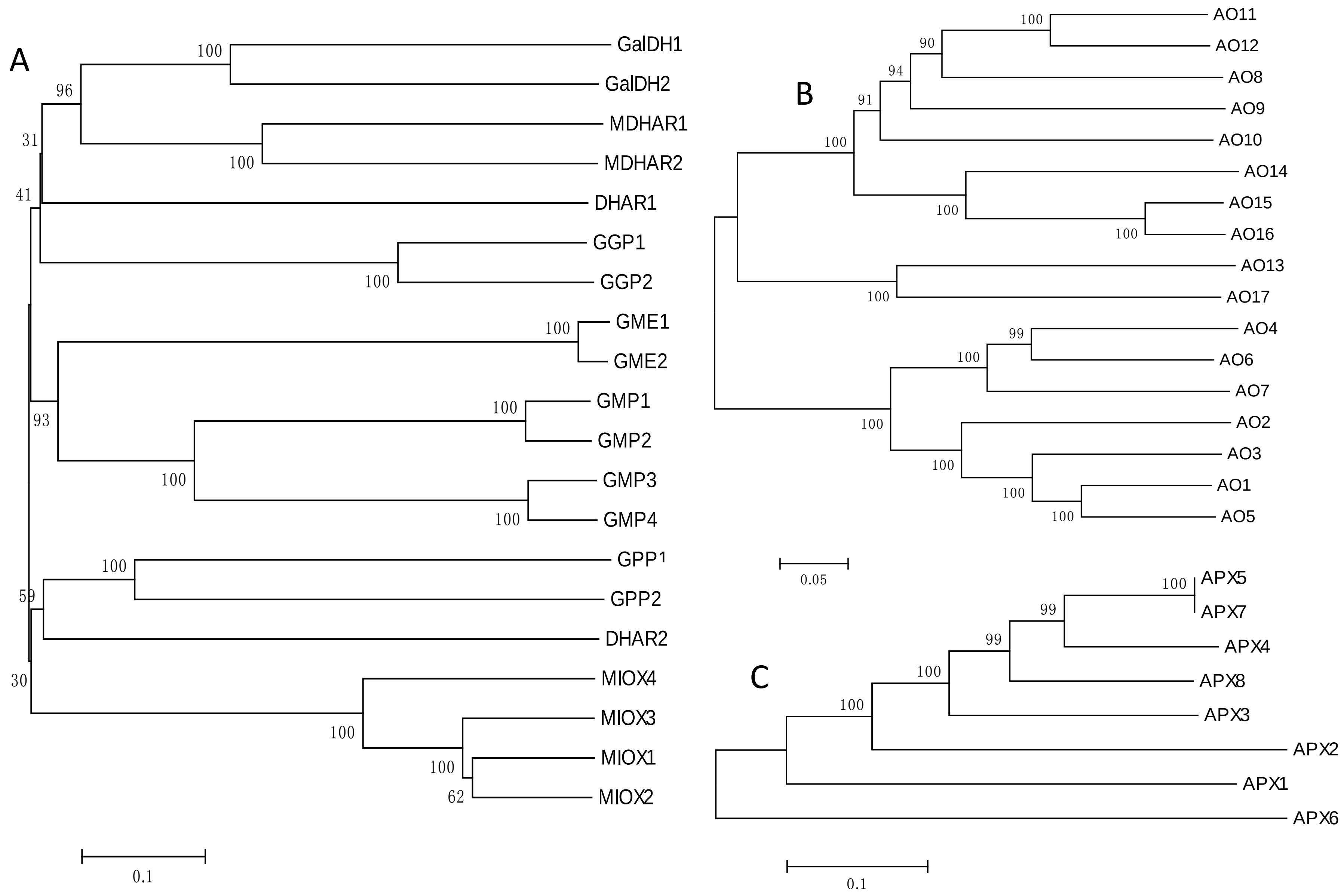

Supplemental figure Maximum likelihood phylogeny of the genes involved in AsA biosynthetic, degradation, and recycling. (A) AsA biosynthetic and re cycling gene families **(B)** ascorbate oxidase family **(C)** ascorbate peroxidase family
